# Recent Applications of Dual-Stimuli Responsive Chitosan Hydrogel Nanocomposites as Drug Delivery Tools

**DOI:** 10.3390/molecules26164735

**Published:** 2021-08-05

**Authors:** Pumtiwitt C. McCarthy, Yongchao Zhang, Fasil Abebe

**Affiliations:** Department of Chemistry, Morgan State University, Baltimore, MD 21251, USA

**Keywords:** chitosan, stimuli-responsive, biomaterials, nanocomposites

## Abstract

Polysaccharides are a versatile class of macromolecules that are involved in many biological interactions critical to life. They can be further modified for added functionality. Once derivatized, these polymers can exhibit new chemical properties that can be further optimized for applications in drug delivery, wound healing, sensor development and others. Chitosan, derived from the N-deacetylation of chitin, is one example of a polysaccharide that has been functionalized and used as a major component of polysaccharide biomaterials. In this brief review, we focus on one aspect of chitosan’s utility, namely we discuss recent advances in dual-responsive chitosan hydrogel nanomaterials.

## 1. Introduction

Polysaccharides exist in various locales in microorganisms, animals and plants and serve critical functional roles. For example, polysaccharides such as cellulose (found in plants) and chitin (found in invertebrate exoskeletons) provide important structure to these organisms [1,2]. Naturally occurring polysaccharides provide researchers building blocks that can be expanded on for the development of new polysaccharide-based biomaterials [3]. Introduction of new chemical functional groups to polysaccharides can provide an additional layer of chemical diversity to these structures. This review focuses on discussion of polysaccharides functionalized to promote stimuli-responsiveness.

Stimuli-responsive polymers, also called smart or intelligent polymers, are a new family of polymers that respond to environmental signals or stimuli by altering their physical and/or chemical properties. The most common stimuli are temperature, pH, light, the electric field, and specific molecules. Promising applications of these polymers are predicted and many have been developed recently developed [4]. Among these, polysaccharide-based stimuli-responsive materials are of particular interest and have potential utility due to their non- to low toxicity, biocompatibility, and biodegradability, in addition to their abundance. These biomaterials have applications to controlled drug-delivery, wound healing, and tissue regeneration. For the purposes of this brief review, the focus will be on chitosan-based stimuli-responsive materials.

## 2. Chitosan Structure

**Figure 1:**
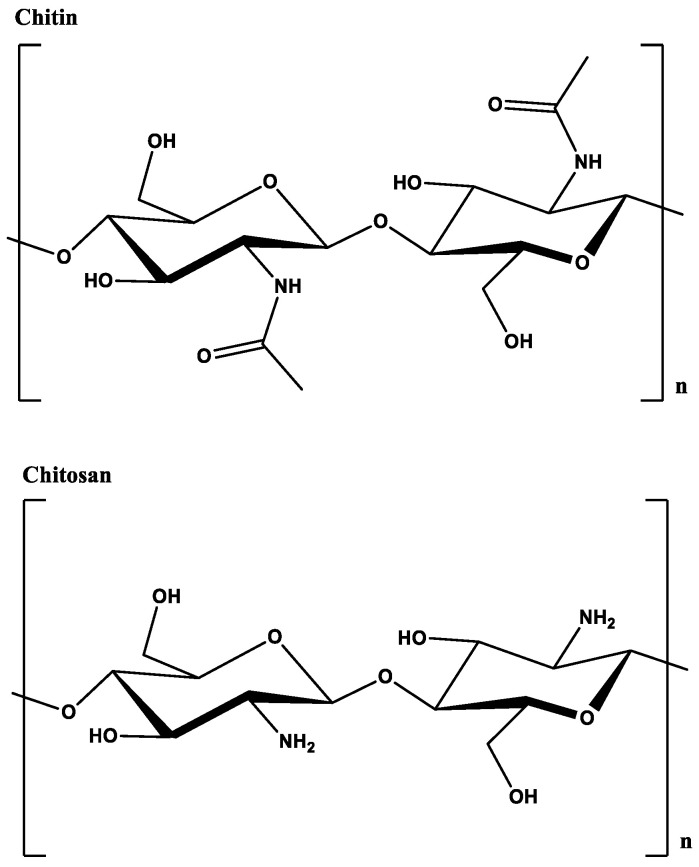
Structure of chitin and chitosan.

Chitin (Figure 1), poly (β-(1→4)-*N*-acetyl-D-glucosamine), is the second most abundant polysaccharide found in nature [5]. The polymer can be found in the exoskeleton of crustaceans, insects, and fungi cell walls. There is a large amount of hydrogen bonding within chitin which makes it insoluble. Partial deacetylation of chitin leads to chitosan, a linear polysaccharide containing a random distribution of β-(1→4)-linked D-glucosamine (missing the *N*-acetyl group) and *N*-acetyl-D-glucosamine (with the *N*-acetyl group). Chitosan can be produced from chitin by the addition of strong base or enzymatically using chitin deacetylases [6]. The amine groups render a pH-dependent solubility to chitosan: it is soluble when the pH is below the pKa of ~6.5 and becomes insoluble when the pH is higher. This property of chitosan and derivatives has been extensively used to fabricate signal-directed, on-demand hydrogel films for various applications [7,8,9,10,11]. Chemical modifications of chitosan to facilitate grafting to polymers have been reviewed elsewhere recently [12].

In addition, recent reviews have covered individual types of stimuli-responsive chitosan (redox, near infrared, electric field, magnetic, pH, temperature) for a variety of biomedical applications [13]. Table 1 lists some recent articles with other types of stimuli and chitosan-based nanocomposites. This brief review focuses only on recent applications that incorporate dual stimuli responsive chitosan for controlled drug delivery using nanocomposites.

## 3. Stimuli-Responsive Chitosan

### 3.1. Utility of pH and Temperature as Individual Stimuli

The pH-dependent protonation and de-protonation of chitosan amines has been exploited to introduce pH-responsive properties. Protonation of amines at low pH results in swelling of the cationic chitosan network and uptake of negatively charged species in the hydrogel, while deprotonation at high pH leads to release of the absorbed species [56]. Extensive studies on chitosan-based, pH-responsive materials have been reported. For example, swelling of chitosan-based hydrogels is highly pH-dependent and reversible and depends greatly on the crosslinking [57].

Temperature is one of the first and most widely used external stimuli, and thermo-responsive chitosan polymers are fabricated by grafting or copolymerizing thermo-responsive units or polymers, such as poly(N-isopropylacrylamide) or PNIPAM [58]. PNIPAM-based hydrogels are fully hydrated at room temperature; however they undergo phase changes when heated above the lower critical solution temperature (LCST) of 32 °C where they lose most of their volume and become dehydrated. Since its LCST is close to human physiological temperature, PNIPAM and derivatives have been widely investigated for possible applications in controlled drug delivery and tissue engineering.

As discussed, chitosan-based hydrogels due to the chemical properties of chitosan are inherently pH responsive. With introduction of PNIPAM-based derivatization, chitosn-based hydrogels have added temperature responsiveness. For biomedical applications, these two stimuli are commonly used because of their drug-release reactivity at near physiological conditions. While physiological pH is 7.2–7.4, in certain disease states cells are slightly more acidic. Some recent examples of pH and temperature as individual stimuli in chitosan-based hydrogel materials are discussed below.

In recent work on pH-responsive hydrogels by Omidi et al., injectible hydrogels were synthesized from the cross-linking of graphene, chitosan, and cellulose nanowhiskers using Schiff base chemistry and a synthetic dialdehyde [59]. The resulting hydrogels were independently loaded and co-loaded with the anticancer drugs doxorubicin and curcumin. Investigation of the in vitro release of the drugs in phosphate buffer show a pH dependent release. Decreasing the pH from 7.4 to 5.4 produced 28% more release of doxorubicin and 30.1% more release of curcumin from independently loaded hydrogels. When both drugs are loaded within the same hydrogel, there is a 28.6% increase in release of curcumin when the pH is decreased from 7.4 to pH 5.4. For doxorubicin, this increase is 38%. The authors propose that in their system the cross-linking of the imine bonds becomes weaker while repulsive electrostatic interactions among the protonated amino groups increase leading to increased drug release with pH. To further confirm the compound’s ability to form a hydrogel, the authors injected the compound into the skin of rats and observed rapid gel formation. Taken together with the fact that the hydrogels exhibited antibacterial activity against *Streptococcus aureus* and *E. coli*, the work by Omidi et al. is a promising avenue for further optimization.

Rasool and colleagues prepared chitosan/poly(*N*-vinyl-2-pyrrolidone) (PVP) based pH sensitive hydrogels for the controlled release of the wound healing drug silver sulfadiazine [60]. Three different configurations of the gels were synthesized each containing a different amount of PVP (0.25, 0.50 and 0.75 g respectively). The configuration with 0.50 g PVP exhibited the most desired swelling behavior and was used to test the release of Ag-sulfadiazine in phosphate buffer saline in vitro. These gels exhibited fast initial release of 35% within the first 10 min followed by continuous release over a period of 1 h with 91.2% of the drug finally being released.

The pH responsive behavior of chitosan can be coupled with protein-type structures to produce materials with expanded functionality for drug release. In a brief example, Xu and colleagues, recently synthesized a series of stimuli responsive composite hydrogels based on silk fibroin protein (SF) and chitosan (CS), cross-linked by EDC/NHS [61]. The internal structures of the hydrogels included macroporous and mesoporous architecture. The in vitro drug release rate demonstrated pH-responsive behavior which is an indication of this material being potentially useful for controlled drug release. A recent review expands on these types of composite materials [62].

Fang et al. developed temperature-sensitive hydrogels composed of poly(*N*-isopropylacrylamide) (PNIPAAm) alone, chitosan grafted to PNIPAAm (CPN) and CPN grafted to hyaluronic acid (CPNHA) [63]. Grafting was performed using EDC/NHS chemistries. The transition temperature among all three gels were within the same physiologically compatible range as assessed by UV/Vis, differential scanning calorimetry and viscosity (PNIPAAm, 29.9–32.0 °C; CPN, 31.1–32.8 °C; CPNHA, 30.5–32.7 °C). The three hydrogels were investigated for their ability to provide controlled release of three types of drugs nalbuphine, indomethacin, and a nalbuphine prodrug in vitro. Each hydrogel had different release characteristics depending on the type of drug. In all cases, the control had the highest release percentage, indicating that grafting of the chitosan or chitosan-hyaluronic acid decreased the release from the hydrogel over the time period observed (60 h). For nalbuphine, excess water was removed and the release of the drug from the hydrogel aggregates were observed. Under these conditions the trend was reversed, CPN had greatest release followed by CPNHA then PNIPAAm. An in vivo test was performed to determine the duration of drug release of the pain reliever nalbuphine after intravenous injection in rats. CPNHA provided the longest pain relief for the rat (~4 h) while CPN provided ~3–4 h. PNIPAAm provided no benefit over the control.

A drug delivery system by Zhou et al. made from chitosan (CS) and αβ-glycerophosphate (αβ-GP) exhibited a thermoresponsiveness that was dependent on physicochemical characteristics such as concentration, molecular weight, and degree of deacetylation of chitosan [64]. The optimal percentage of deacetylated chitosan in the formulations was found to be 75%; at percentages higher and lower than this there was no gel formation. Accordingly, the optimal molecular weight was 1360 kDa. In terms of temperature, gel formation was observed to occur at 37 °C which increases the potential for its use as an injectible. Release rate kinetics were observed for both adriamycin (a hydrophilic model drug) and 6-mercaptopurine (a hydrophobic model drug) respectively from CS-αβ -GP hydrogels created with optimal conditions. Adriamycin was released 70% over 24 h which was slower than release of of 6-mercaptopurine over the time period (nearly 50%). This work suggests this hydrogel could be ideal for sustained release of hydrophilic drugs.

### 3.2. Dual-Stimuli Responsive Chitosan

Strategies that introduce two or more variety of stimuli in one construct are advantageous because they offer synergistic control over the release of the compound of interest. For the sake of this review, our focus is on recent literature examples that combine dual stimuli.

#### 3.2.1. pH and Temperature-Responsive Release of Bioactive Agents

**Figure 2:**
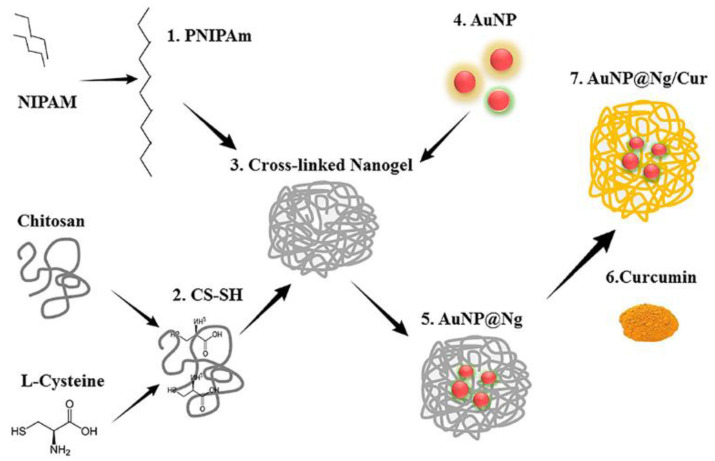
Schematic of production of a cross-linked nanogel by Howali et al. (From [47]).

**Figure 3:**
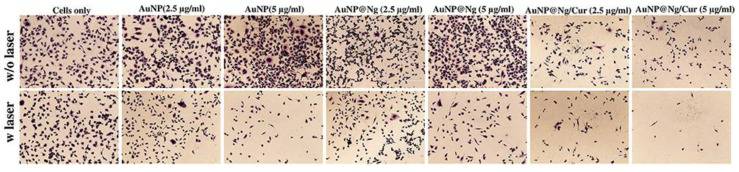
Nanogel induced cell death after irradiation (modified from [59]).

Chitosan is particularly well-suited for action at low pH and cancer cells are generally more acidic than normal cells. Nanoparticles are well suited for drug delivery as they can be modified with molecules to allow for targeting to cancer cells [65]. When magnetic nanoparticles (such as Fe_3_O_4_) are incorporated into chitosan-based hydrogels, the hydrogels became responsive to the external magnetic field and could deliver therapeutic drugs with superior targeting and prolonged retention while avoiding excretion [66]. Recent work by Howaili et al. utilizes a nanogel of cysteine-modified chitosan grafted to PNIPAM and gold nanoparticles (AuNPs) (Figure 2) [67]. Curcumin, a bioactive compound with anti-cancer activity [68], was loaded within the nanogel. At physiological pH and room temperature, there were low levels (20%) of curcumin release from the hydrogel over 72 h. At pH 5.5 and 37 °C, this increased to 80% over 72 h. The use of nanoparticles allows for IR induced irradiation of the nanogels which enhances thermo-responsiveness [58]. This curcumin loaded-nanogel entered cells and were toxic against a breast cancer cell line. In this work, cells from an MDA-MB-231 breast cancer cell line were exposed to gold nanoparticles, the nanogel, and the curcumin-loaded nanogel respectively in the absence or presence of NIR laser irradiation at 808 nm (Figure 3). However, the most cell death was observed for the curcumin loaded-nanogel exposed to laser irradiation in dose-dependent manner indicating this combination of pH and NIR light, led to release of curcumin and induced cell death (Figure 3).

In another example of NIR/pH/temperature stimulating compound release, single-walled carbon nanotube–chitosan–PNIPAM (with PEGylation) composites were investigated by Qin and coworkers [69]. An oleic acid-modified chitosan copolymer (CS-OA) was first used to surround the carbon nanotube. The lipophilic side-chains were interacting with the carbon nanotube while the hydrophilic chitosan backbone was exposed to the aqueous environment. This modified nanotube (CS-OA@CNT) was further modified with PNIPAM and a polyethylene glycol (PEG) based crosslinker to create a nanogel. Doxorubicin was loaded within the nanogel with an efficiency of 90% and loading capacity of 43.3 wt % as determined in vitro. This nanogel delivery system exhibited increased release with NIR irradiation (808 nm), low pH (5.0) and higher temperature (40 °C). Researchers also assessed the effects of drug release in vivo as a function of doxorubicin-CS-OA@CNT cytotoxicity using HeLa cells with higher cell killing observed in the presence of NIR irradiation. Confocal microscopy of the cells confirmed cellular uptake of the nanoparticles.

Jaiswal and coworkers incorporated Fe_3_O_4_ magnetic nanoparticles with PNIPAM-modified chitosan to create nanogels which were then loaded with doxorubicin. Loading of doxorubicin in the nanogel was estimated to be ~60% [70]. In vitro release studies with the nanocomposite over a 1 h period in buffer showed the most release (90%) of drug at a temperature of 42 °C (the LCST of the nanocomposite) and low pH compared to 43% at 37 °C. The ability of the nanoparticle-gel system to induce cell death in human breast (MCF-7) and cervical carcinoma (HeLa) cell lines after 24 h incubation and subsequent application of a magnetic field was assessed. The system was cytotoxic to both cell lines. It was determined that the combination of the magnetic field, low pH and high temperature led to 85% increase of cell death in HeLa cells.

Chitosan-based hydrogels with in situ production of nanoparticles have also been investigated for release of bioactive compounds [71]. *N*-Succinyl-modified chitosan was linked to zinc oxide (ZnO) nanoparticles and curcumin as a potential drug delivery system [72]. The in vitro kinetics of release of curcumin in PBS at pH 7.4 vs. pH 5.2 over 1 h was 20% vs. 45% indicating more curcumin release in a shorter release amount of time at lower pH. After 16 h, this release increased to 95% at low pH vs. 80% at higher pH. This composite system was also able to induce more apoptosis in MDA-MB-231 cells compared to the same concentrations of free curcumin. An added benefit of these nanoparticles was the increased antibacterial activity against *Staphylococcus aureus* and *Escherichia coli*. The minimum inhibitory concentrations (MIC) and minimum bactericidal concentration (MBC) were dramatically lowered compared to the free components. For example, the MIC and MBC for free curcumin against *Staph. aureus* was 250 µg/mL and 500 µg/mL respectively. With the nanocomposite the MIC value was lowered to 10 µg/mL and the MBC value was 50 µg/mL.

A novel method for in situ nanoparticle formation was put forth by Hosseinzadeh et al. which used Reversible Addition−Fragmentation Chain Transfer (RAFT) polymerization to create a chitosan hydrogel composite with magnetic Fe_3_O_4_ nanoparticles [73]. In their method, the researchers first incorporated *N*-phthaloyl protecting groups onto the amino groups of chitosan. A RAFT agent was added to the hydroxyl groups of chitosan and this *N*-phthaloyl chitosan-RAFT was used for esterification to the hydroxyl side chains of the magnetic nanoparticles. To synthesize the magnetic nanocomposite, copolymerization was performed using acrylic acid and NIPAM. The nanocomposite was loaded with doxorubicin. This nanogel exhibited a pH and temperature-controlled release of doxorubicin over 24 h. In buffered solutions at low pH and higher temperature (45 °C), ~90% (pH 2) and ~78% (pH 5.0) of drug release was observed. At this temperature, ~40% was released at pH 7.4. These values are much higher than those observed at 25 °C [73].

Dual pH and temperature-responsive nano-composite double network PNIPAM/clay/carboxymethyl chitosan (CMCTs)/genepin (GP) hydrogels with high mechanical strength were synthesized through a one-pot reaction in which UV light induced free radical polymerization [74]. The adsorption/desorption kinetics of the hydrogel was dependent on the PNIPAM, clay, CMCTs, GP and hydrogel ratio. Release behaviors of the hydrogels were investigated using acetylsalicylic acid (ASA) as a model drug. The highest release percentage of the hydrogels decreased with an increase of CMCTs, GP or clay. This was most likely due to increased cross-linking. With optimal ratio of components and under optimal conditions, ~89% cumulative release of ASA was observed.

A chitosan-based hydrogel using tris(2-(2-formylphenoxy)ethyl) amine as a cross-linking gelator was recently developed by Karimi and others [75]. Hydrogel formation was a result of the Schiff base formation between chitosan amine groups and aldehyde groups of the crosslinker. Optimal swelling ratios were observed at low pH and high temperature. Release of metronidazole from the pH- and thermo-responsive hydrogels were investigated under pH conditions to mimic the gastric juices (pH 1.8) and interstitial fluid (pH 6.8). The different swelling behaviors at various pH values are responsible for the differences of the drug release in the medium. As expected, drug release increased with decreased gelator (14% vs. 10%). Under both pH extremes, at least 90% of the drug was released over 600 min.

#### 3.2.2. Other Examples of Dual-Responsive Release of Bioactive Compounds

##### Redox- and pH-Responsive

Redox-responsive drug carriers have been fabricated by crosslinking chitosan-based composites with disulfide linkages. At normal physiological pH, a high concentration of glutathione (GSH) does not cleave the SS bond and the drug is not released; however under acidic conditions GSH quickly cleaves the SS bond and the drug is released [76].

**Figure 4:**
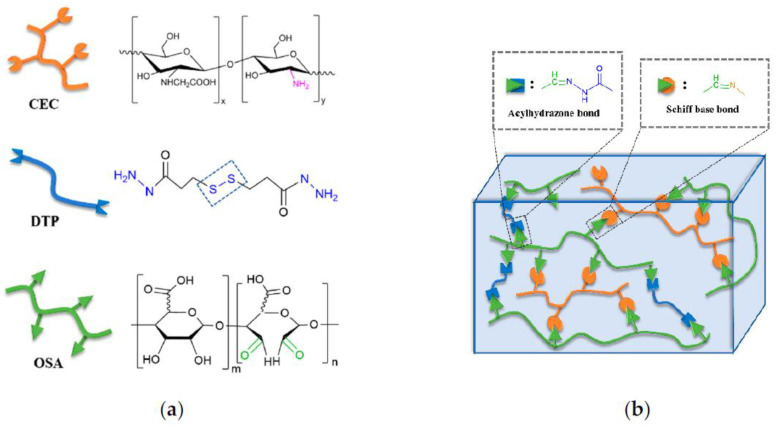
(**a**) Structures and illustrations of carboxymethyl chitosan (CMC), 3,3′-dithiopropionic acid dihydrazide (DTP) and oxidized alginate (OSA); (**b**) schematic of crosslinking strategy (modified from [80]).

These conditions thus rely on pH and redox poise of the target cell. In recent work, Ding and colleagues have created a novel hydrogel system in which a pH sensitive allyl-modified chitosan (OAL-CS) is disulfide-linked to thermo-responsive modified NIPAM using thiolene “click” chemistry and RAFT copolymerization [77,78]. This hydrogel system affords precise control of the OAL-CS/NIPAM ratio. Ratios of 20:1, 10:1, and 5:1 were investigated in this study. Hydrogels with the highest ratio (20:1) gave the optimal swelling ratio and the gels showed biocompatibility in vivo and in vitro. Ding et al.’s work provide a new potential platform for drug delivery. Recently, Chen et al. developed high-performance hydrogels through use of known biocompatible polysaccharide derived polymers, carboxymethyl chitosan (CMC), and oxidized alginate (OSA). Combined with the crosslinking agent, 3,3′-dithiopropionic acid dihydrazide (DTP) the resulting CMC-OSA-DTP hydrogels were injectible, biocompatible and possessed the ability to self-heal (Figure 4) [79]. Future development of this hydrogel could be geared towards use as a drug delivery system.

##### Electro- and pH-Responsive

When placed in an electric field, hydrogels formed from charged polymer networks may experience “bending” towards one of the electrodes. Chitosan-based hydrogels have been shown to possess this electric field responsiveness which may be used as soft electro-actuators, tissue engineering scaffolds, and controlled release drug delivery systems. Liu et al. fabricated homogeneous polyelectrolyte complex (PEC) hydrogels from positive chitosan and negative carboxymethylcellulose [29]. When an external DC voltage was applied to the salt solution in which the hydrogel strip was placed, movement of the ions caused uneven distribution of the counter ions near the hydrogel surface, resulting in different extent of hydration/swelling of the two sides of the hydrogel strip, and bending of the strip. The authors found that between 9 and 27 V the bending angle and response time depended approximately linearly on the voltage. Electro-responsive release may also be achieved by applying a current.

Kiaee and colleagues recently developed an electric field responsive and pH-controlled hydrogel containing chitosan loaded nanoparticles enveloping a model compound (Fluorescein isothiocyanate-dextran (FITC)) to mimic drug release [80]. The author’s propose this as a potentially new avenue for control of compounds at wound sites. The system consists of a PEG-diacrylate/laponite hydrogel containing chitosan particles, an electronic driver, and microelectrodes on a flexible substrate. In their work, application of the electric field increases the pH in a rapid and controllable way. The voltage when applied in an on/off pattern of 2 s application of 4 V leads to an increase of pH from 6 to 12 in 1 s and the pH returns to 6 after 2 s. Release studies in vitro under these conditions show that they were able to obtain 100% of release of the FITC compound. The pH and electro-responsive hydrogel was also found to have good biocompatibility. In an in vivo wound scratch assay, there was no decrease in the ability of cells to heal in the presence of the wound patch and in the absence or presence of the magnetic field (2.5 and 4 V, 5 min). In addition, there were no differences apparent in the absence or presence of the nanogel using live/dead cell assays. Thus this gel could be further optimized for use in release of drug compounds at wound sites [80].

A pH and electroresponsive system consisting of a chitosan hydrogel with embedded mesoporous silica nanoparticles (MSNs) deposited on a titanium plate was evaluated for drug release properties [81]. Ibuprofen was used as a model drug and its release from MSNs/chitosan hydrogel films were studied. Release was dependent on the pH (with neutral and basic conditions more favored than acidic) and the voltage applied to the titanium plate. Spontaneous release of ibuprofen from the hydrogel was measured in 0.9% NaCl solution (at 0 V) and compared to the release observed after the application of negative voltage at the cathode (−1.0, −3.0 and −5.0 V). The fastest release kinetics was observed at −5.0 V. These results suggest the potential interplay of complex IB-MSNs/chitosan hydrogel films with titanium implants as a method for controlled drug delivery.

##### Light and Temperature as Responsive Stimuli

In a recent study, Srinivasan et al. reported on the production and characterization of IR820-chitosan conjugates and showed its multifunctional potential: the conjugate could act as an imaging agent as well as induce cancer cell killing upon irradiation. The conjugates were able to generate heat upon exposure to a laser at 808 nm and the hyperthermic conditions led to cell growth inhibition in cancer cell lines [82]. Work by Shin and colleagues produced a thermo-responsive nanocomposite hydrogel which contained crosslinked chitosan and PNIPAM nanogels for controlled drug delivery [83]. Wang et al. created a novel light-and thermoresponsive composite through use of methacrylate modified chitosan and PNIPAM. A photothermal carbon (PTC) was also embedded within the network. In the presence of doxorubicin, UV light was used to induce crosslinking (through the PTC and double bonds of the methacrylate groups) and doxorubicin would be loaded into the gel. To induce drug release, a near IR laser (808 nm) would trigger an increase in temperature leading to decreased volume of the gel and drug release. Release of doxorubicin was observed to be 40 times higher after NIR irradiation [84]. Other light-responsive drug delivery systems have also been reported [85,86].

##### Glucose and pH Responsive Hydrogels

Among literature reports of dual-responsive hydrogels in which small molecules are one of the stimuli, glucose is the most prevalent small molecule used. To confer glucose sensitivity phenylboronic acid can be incorporated into a system. Phenylboronic acid has been shown to form stable covalent bonds with diols [87]. In proof-of-concept work by Li et al., a dually responsive injectable hydrogel was used as a doxorubicin-carrier. Tumor cells, due to their increased metabolic function, are estimated to have 3–10 fold higher glucose levels than normal cells [48]. Therefore, a potential drug carrier targeting cancer cells would have more interactions with glucose than a typical cell. The work of Li and colleagues sought to capitalize on that by creating a hydrogel that combined a pH trigger and a glucose responsive phenylboronate ester. Using a combination of phenylboronic acid modified chitosan (CSPBA) and oxidized dextran (Oxd), the system also contained both a pH- responsive imine bond that was stable at neutral/physiological pH and labile at slightly acidic conditions. Using the lowest grafting ratio and least oxidized dextran, the highest release of doxorubicin in vitro was achieved after 12 h. At pH 7.4, 52% of the drug was released compared to 70% when the pH was decreased and 61% with adding glucose at pH at 7.4. In higher crosslinking hydrogels, this release was less. In vitro tests of hydrogel formation showed that the gels formed only at the site of injection. For in vivo studies, test of cell viability were performed using L929 fibroblast cells. The cells were grown within the hydrogel and the cell shape and morphology indicated good cell viability for three days. In cell proliferation studies using the same type of cells, more cell proliferation was seen with hydrogels that had a lower polymerization percentage. The authors proposed perhaps larger pore size allows for more nutrient exchange. Compared to the hydrogels with more crosslinking they observed the same or slightly less cell proliferation.

Glucose/pH responsive gels have also been used to target diabetes. The disease results in typically elevated glucose levels because of not enough insulin being made or improper function of insulin (type II) or a complete lack of insulin synthesis (type I). Glucose responsive nanocomposites have been investigated to provide a way to modulate drug release based on glucose concentration. In work by Zhao and colleagues, a pH and gluco-responsive carrier was made by use of graphene oxide (GO) as a nanocarrier for the model drug (bovine serum albumin, BSA) [54]. Cationic chitosan (HTCC: glycidyltrimethylammonium chloride-grafted chitosan) was used with GO-BSA to form a complex. This chitosan served as the pH responsive component of the hydrogel. The glucose responsive moiety in this system was glucose oxidase, an enzyme responsible for glucose oxidation. The authors studied the effects different wt % of GO-BSA (0%, 0.5%, 1.0%, 1.5%, 2.0%) had on drug release. The outcomes of the work indicated that the HTCC/BSA had good drug release but there was a slightly more glucose specific effect for the 2.0 wt % material.

In one of the most comprehensive examples of the application of these dual stimuli, Gu and coworkers developed novel injectable microgels that were pH and glucose-responsive [47]. These gels provided a targeted insulin release and were tested in mouse models of type I diabetes. The hydrogels contained chitosan microgels inside which nanoparticles housing glucose oxidase and catalase were present. Glucose oxidase performs the reaction that oxidizes glucose to gluconic acid during which there is concomitant production of hydrogen peroxide. Catalase acts to convert hydrogen peroxide to oxygen and water. Both enzymes were covalently attached to the nanoparticle which was housed within the chitosan microparticle. In vivo studies were performed to determine the ability of these microgels to lower the higher glucose levels seen in diabetic mice. Three versions of the microgels were tested: a microgel containing insulin with no enzymes, one containing enzymes and no insulin and one containing insulin and enzymes. Remarkably the microgel containing insulin and enzymes, lowered the glucose levels to normal levels within in two hours and was the only formulation to keep these levels consistently low for an additional 10 h.

#### 3.2.3. Stimuli-Responsivity in Combination with Biomolecular Targeting

Up to this point our discussion has focused on the combination of two types of stimuli. However, chitosan-based nanomaterials have been engineered to react or even incorporate specific biomolecules for targeting at specific cellular sites. For example, Liu and coworkers sought to create a responsive insulin-containing nanocomposite gel that when delivered orally, could withstand the acidity of the gastric juices and still be able to deliver the insulin payload [88]. A hydrogel surrounded an insulin/heparin sulfate aggregate encased nanoparticle which was coated with chitosan. After administration to diabetic mice, there was more bioavailable insulin in mice that received insulin orally via the pH and amylase-responsive gel versus not indicating that this method could provide new means to enhance stability.

Phosphorylated chitosan was found to have adjuvant properties and enhance the immunogenicity of vaccines in a proof-of-concept study in work by Wei and others. Phosphorylated chitosan has enhanced water solubility compared to underivatized chitosan but still experiences gelation at pH lower than neutral. Use of phosphorylated chitosan as a delivery system during ovalbumin injection led to an increase in antigen specific immune responses in mice [89].

In recent work, Yu and co-workers constructed stimuli-responsive three-dimensional cross-linked hydrogel system containing carboxymethyl chitosan (CMC) and poloxamer composed of a poly (ethylene oxide)/poly (propylene oxide)/poly (ethylene oxide) (PEO–PPO–PEO) block copolymer [90]. Using the drug nepafenac (NP) as a model drug, the controlled drug release behavior of these hydrogels was investigated. The drug release rate reached a maximum at 35 °C and pH 7.4. These conditions provide the highest swelling ratio and correspondingly the largest microchannels. The results indicate the hydrogel could be used as a pH-temperature-responsive drug delivery system that can be targeted to the human eye.

A poloxamer-chitosan (PLX-CS) and carbopol-HPMC-alginate (CP-HPMC-SA) hydrogel systems was characterized for its potential to enter the blood brain barrier. Appropriate gelation time, temperature, and pH were followed to induce release of vinpocetine. This drug is a preventative therapy for those who suffer from degenerative diseases that affect the brain and nervous system. Both hydrogel formulations loaded with vinpocetine, were administered to rats intranasally and the blood and plasma levels were compared to levels of the drug at these sites after injection. Remarkably, the hydrogels had at least 2× more drug found in the brain compared to what was seen for the injection. Inversely, the hydrogels had less drug present in the plasma compared to injection. This provides proof-of-concept data that indicates these hydrogels were able to bind to the mucosal layer and enter the brain [91].

## 4. Future Applications of Dual-Responsive Chitosan

Future efforts in dual responsive chitosan hydrogel technology, may be inclusion of compounds that also add labeling functionality. Huang and co-workers used mesoporous silica grafted with PNIPAM-chitosan and the luminescent decatungtoeuropate. This pH and temperature sensitive composite exhibited high release of doxorubicin (73%) over a 24 h period at 42 °C. The red luminescence from decatungtoeuropate could be seen using at room temperature and 42 °C. The composites were biocompatible with low toxicity. Doxorubicin-loaded composites were shows to kill HeLa cells in a dose-dependent manner (up to 50 µg/mL) [92]. These nanocomposites could open the door to new types of MRI imaging agents.

While the focus of this review has been a description of dual-responsive chitosan in drug-delivery, the stimuli-responsivity of chitosan could be applied tangentially to chitosan’s well-known role in the capture of heavy metals. There have been several recent publications in which chitosan-grafted nanoparticles and nanocomposites [93,94,95,96,97,98,99,100,101,102,103,104,105,106], gels and resins [107,108,109,110,111,112] were used to adsorb toxic metals such as chromium, copper, lead and cadmium from aqueous solutions. Dual-responsive polysaccharide derivatives that make use of combined strategies of visual metal detection using turn-on metal sensing [113,114,115] and stimuli-induced hydrogel formation [116] may add even more functionality to chitosan and other novel polysaccharide structures that have recently been shown to capture heavy metal [117]. A recent study indicates such a method could be viable as metal ions were used as one form of stimuli for hydrogel formation [118].

## 5. Conclusions

In closing, we have focused on selected examples that highlight the benefits of chitosan hydrogel-based multi-responsive nano-composites. These biomaterials have good compatibility, the ability to adsorb and release drugs/model drugs in vitro and in vivo, and offer more control over hydrogel formation. Induction of more than one mode of responsiveness increases control over gel formation and subsequent release of the bioactive compound. This discussion has focused on describing results of recent advances in dual-responsive hydrogel nanocomposites including pH/temperature, pH/redox, pH/electric field, temperature/light, and pH/glucose. The work presented in this review illustrates the balance that must be struck between functionalization being added to chitosan or the grafted molecule and the degree to which this grafting takes place. The physicochemical properties of the hydrogel can be affected by these factors which in turn affects the release of the desired drug from the designed gel.

## Figures and Tables

**Table 1:** Recent literature references for stimuli-responsive chitosan based-nanocomposites.

Stimuli	References
Redox	[14,15,16,17,18,19,20,21,22,23]
Electric field	[24,25,26,27,28,29,30,31,32]
Light	[33,34,35,36,37,38]
Magnetic field	[39,40,41,42,43,44,45]
pH	See text
Temperature	See text
Small molecule (Glucose)	[46,47,48,49,50,51,52,53,54,55]
